# Graphene Oxide Thin Films with Drug Delivery Function

**DOI:** 10.3390/nano12071149

**Published:** 2022-03-30

**Authors:** Alexandra M. L. Oliveira, Mónica Machado, Gabriela A. Silva, Diogo B. Bitoque, Joana Tavares Ferreira, Luís Abegão Pinto, Quirina Ferreira

**Affiliations:** 1 Instituto de Telecomunicações, Avenida Rovisco Pais, 1049-001 Lisbon, Portugal; monica_mac@hotmail.com; 2 iNOVA4Health, CEDOC Chronic Diseases Research Centre, NOVA Medical School, Universidade Nova de Lisboa, Campo Mártires da Pátria 130, 1169-056 Lisboa, Portugal; gabriela.silva@nms.unl.pt (G.A.S.); diogo.bitoque@nms.unl.pt (D.B.B.); 3 NOVA Medical School, Faculdade de Ciências Médicas, Universidade Nova de Lisboa, 1169-056 Lisbon, Portugal; 4 Ophthalmology Department, Centro Hospitalar Universitário de Lisboa Norte, 1649-035 Lisbon, Portugal; joana.t.ferreira@chln.min-saude.pt (J.T.F.); l.pinto@campus.ul.pt (L.A.P.); 5 Visual Sciences Study Centre, Faculty of Medicine, Universidade de Lisboa, 1649-028 Lisbon, Portugal

**Keywords:** graphene oxide, drug delivery, polyelectrolyte multilayer films, layer-by-layer

## Abstract

Graphene oxide has been used in different fields of nanomedicine as a manager of drug delivery due to its inherent physical and chemical properties that allow its use in thin films with biomedical applications. Several studies demonstrated its efficacy in the control of the amount and the timely delivery of drugs when it is incorporated in multilayer films. It has been demonstrated that oxide graphene layers are able to work as drug delivery or just to delay consecutive drug dosage, allowing the operation of time-controlled systems. This review presents the latest research developments of biomedical applications using graphene oxide as the main component of a drug delivery system, with focus on the production and characterization of films, in vitro and in vivo assays, main applications of graphene oxide biomedical devices, and its biocompatibility properties.

## 1. Introduction

**Figure 1:**
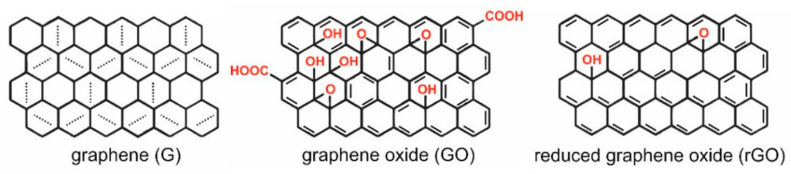
Graphical representation of the molecular structures of different graphene-based materials. Republished from Ref. [18].

Graphene-based materials have an important role in the development of drug delivery systems (DDS) due to their biocompatibility properties and the easiness with which they can be functionalized with biomolecules. Moreover, it is simple to integrate them in multilayer systems with applications in which controlled release of bioactive molecules is necessary. Graphene is a two-dimensional compound, consisting in a monolayer of aromatic carbon atoms (sp2-bound)/covalently bound, organized in a hexagonal lattice structure [1], forming sheets with the thickness of a single atom [2]. It has interesting mechanical properties, large surface area, and high electrical and thermal conductivities [3], and it can exist in other forms, such as graphene oxide (GO) and reduced graphene oxide (rGO) (see Figure 1), which are more hydrophilic, making them easier to solubilize and disperse in aqueous or polyelectrolyte solutions (such as PBS—phosphate buffered saline) and improving, therefore, their self-assembling properties [4,5]. The high content of groups with reactive oxygen in GO enhances its ability to be functionalized with various materials [1]. It is composed of carboxyl, hydroxyl, and ether groups, allowing it to absorb polar polymers or polar molecules with ease, and, therefore, it is an excellent candidate to form GO/polymer composites [4]. These active groups are ideal for molecule immobilization on the GO surface and make it hydrophilic and a powerful candidate to be used as drug carrier [6]. For instance, carboxyl groups are the main group responsible for GO colloidal stability [7,8,9]. Furthermore, GO has good water and biomedium dispersibility as well as good optical (absorption) and photothermal (conversion) properties [10]. Several functionalization strategies can be used to enhance GO’s characteristics and promote its stability and bioavailability. This functionalization can be achieved, for example, by PEGylation (PEG—polyethylene glycol) to improve biocompatibility, solubility, and stability in physiological conditions, by double oxidation of the graphene to provide electrostatic stabilization, by using a copolymer (e.g., Pluronic F127) to provide steric stabilization, by non-covalent interaction (π-π interactions) with aromatic organic molecules (such as 1-pyrenebutyrate (PB)) to improve aqueous stability, or by using synthetic peptides such as Poly L-Lysine (PLL) to improve its biological activity [11,12,13,14]. The versatility in the functionalization of GO allows its application in several devices. An example is the ability of functionalized GO to cross the blood–brain barrier (BBB), which widens its use in biomedical research, as it overcomes the inability of unstable chemicals or peptides to reach the brain [15,16]. Mendonça et al. [17] also demonstrated the ability of rGO to cause a transitory BBB breakdown and, therefore, cross this BBB into the brain.

GO is also a versatile material to be part of multilayer systems with applications in DDS and biosensors. Usually, the layer-by-layer (LbL) is the elected technique to build these systems. It is based on the alternate adsorption of oppositely charged particles (polyelectrolytes) and has been studied more each day, as these polyelectrolyte multilayer films (PEMf) have the ability to allow the fine-tuning of DDS both in time and space, thus permitting therapeutics to be delivered in situ during a course of several days or even months. In the last years, graphene has been studied as a component of PEMf and has shown promising results either as a nanocarrier or in stabilizing and/or delaying the drug release in this DDS. This article revises the recent experiments on graphene-based DDS, including the techniques usually used to develop multilayered films. Two types of uses are described:

1. GO incorporated in multilayered systems in which it takes the role of carrier, helping to transport the therapeutic agents, making sure that they reach the target before being released, and helping to protect them from early degradation.
2. GO incorporated in multilayered systems in which it acts as a barrier or capping layer with the purpose of delaying and/or controlling the drug release across time, in a precise and stratified manner, with control of the release sequence.

Biocompatibility and recent applications are also addressed.

## 2. Graphene Oxide Multilayer Films

**Figure 2:**
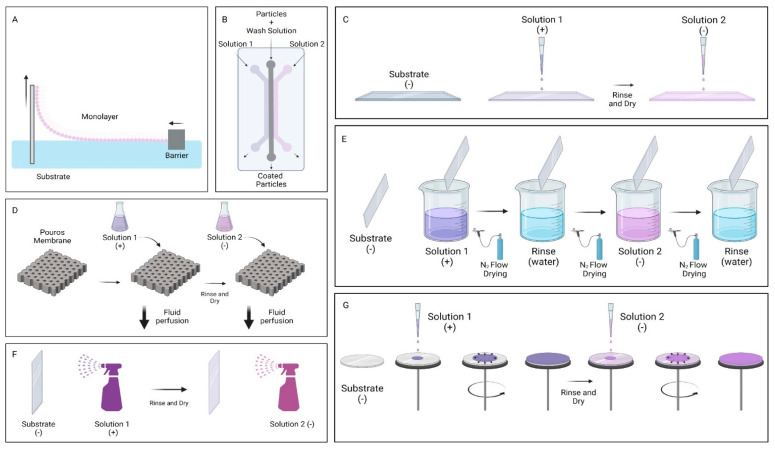
Schematics of the different LbL assembling methods. (**A**)—Langmuir–Blodgett assembly; (**B**)—microfluidics; (**C**)—drop coating; (**D**)—perfusion, (**E**)—dip coating; (**F**)—spray coating; (**G**)—spin coating. Figure created with BioRender.com.

LbL is a versatile technique which allows the incorporation of multiple components culminating in a smart and functional system. It is a simple and cost-effective process that can be used in large-scale production and is eco-friendly, due to the fact that the solutions used are aqueous [9]. LbL is the preferred technique to develop DDS with graphene, and several methods can be used to produce thin films, such as microfluidics [19], perfusion [19], Langmuir–Blodgett assembly [20], spin coating [5], drop coating [21], spray coating [22], or dip coating [4], which is the most common one (see Figure 2).

The major driving forces in LbL multilayered films are electrostatic forces (more common), hydrogen bonding, charge transfer interaction, and covalent bonding [3,23]. LbL films can incorporate functional polymers [24], enzymes [25], small molecules [26], polysaccharides [27], carbon nanotubes (CNT) [28], nucleic acids [29,30], or inorganic nanoparticles (NPs) [31] and can be assembled in a wide range of substrates independently of their size or shape, allowing for the creation of three-dimensional objects covered in these films [3,32]. The fact that LbL films can be deposited on different substrates without altering the properties of the films is very useful, because it allows the testing of different film characteristics, which may require different substrates [9,33], for example, the quartz crystal microbalance (QCM), which requires the films to be assembled in a QCM crystal sensor/lamella [34,35], or the transmission electron microscopy, for which the films need to be deposited onto copper grids [36,37]. This also allows for their application in multiple biomedical devices without fear that their properties will change. Fox example, they can be applied in flexible substrates that can adhere to tissue and deliver different types of drugs [7] or odd-shaped substrates as coatings for biomedical implants, such as cardiovascular stents [38] [9,33]. Furthermore, LbL films containing GO can be used, for example, in tissue engineering [11,39], as carriers for vaccines [40], as antibacterials [41], in cancer therapy [42], or in biomedical imaging [43,44,45].

The incorporation of GO in a multilayered film is a chemistry game; it is necessary to have stable chemical interactions in the interfaces of the layers to avoid interdiffusion of components and to ensure a correct growth of film. Usually, to avoid aggregation or agglomerates on the surface of GO layers, bilayers of charged GO are added. GO has a natural negative charge and can act as a negatively charged component in LbL without further functionalization, and the positively charged GO can be obtained by amine functionalization [9,46,47,48,49]. As a result, there is obtained a thin and smooth bilayer of GO that can be incorporated between polyesters or polymeric thin layers. GO can interact with other molecules via hydrophobic or electrostatic interactions, hydrogen bonding, or π-π stacking. GO can be non-covalently functionalized using LbL to improve dispersibility and avoid aggregation, using, for example, chitosan (Chi)/dextran, Chi/sodium alginate (SA), or protamine sulfate/SA [6,33]. Small molecules, non-polymeric in nature, are more difficult to incorporate into LbL assemblies, because they can penetrate the graphene multilayers, and that can affect the growth of the multilayered system [6].

To start a multilayered film, it is necessary to charge the substrate to enhance the adsorption of the first layer. The most common assembly process is to start with a negatively charged substrate that is usually obtained by piranha solution (composed of sulfuric acid (H_2_SO_4_) and hydrogen peroxide (H_2_O_2_) in the proportion of 7:3, v/v) and oxygen plasma treatment [50] and, therefore, a negatively charged layer. However, it is also possible to invert the order, functionalizing the substrate with positive charges by dipping the substrate in APTES solution ((3-aminopropyl) triethoxysilane) (3 min). Furthermore, substrates should be previously cleaned to remove organic contaminants with piranha solution followed by a rinse with deionized (DI) water [46]. The cleanness and functionalization of substrate is important to ensure the homogeneity of the following layers. Usually, the films exhibit a linear growth; however, this can vary on the first few bilayers, due to interaction with the chosen substrate [9].

The pH of the dipping solutions can greatly affect the resulting film’s thickness and roughness, as it can affect the charge balance between the oppositely charged components and the degree of ionization of weak polyelectrolytes. If the pH and pKa of the dipping solution are near each other, the film’s thickness is increased [46,51]. The use of different or even cross-linked polymers in LbL films allows for the control of the porosity and rate of degradation and dissociation of the films [51].

The growth of a multilayered film can be monitored by several techniques, and it is crucial to perform a stepwise characterization to ensure the homogeneity of each layer, especially when different types of materials are included, as usually happens in a DDS in which graphene can be intercalated with polymers, NPs, drugs, or other biomolecules. Table 1 summarizes commonly used techniques to perform this characterization. Furthermore, a careful monitoring of the drug’s release profile is important. In order to access this, for the most part what happens is the incubation of the films in physiological conditions (generally PBS at 37 °C), and then the PBS solution is analyzed through various methods which might include: UV-visible/fluorescence spectroscopy to evaluate the release kinetics [34,36,52,53,54]; micro-BCA kit to evaluate loading efficiency [55]; measurement of fluorescence spectra for fluorescent molecules [56]; ELISA for release studies of some proteins [57]; and gel electrophoresis and circular dichroism (CD) spectroscopy to evaluate whether proteins released from films maintain their primary and secondary structures [58,59]. Notwithstanding, the erosion of a multilayered DDS can be followed using the same technique that was applied to follow the growth of the film to obtain a detailed interpretation of the drug release behavior.

## 3. Graphene Oxide as Carrier in Drug Delivery

Ideally, nanocarriers must have a size between 30 and 200 nm to be retained in blood vessels. Above this size, they are prone to aggregation in the liver or the spleen, and, below that, they can suffer blood renal filtration, being filtered from the plasma to the urine [8]. When encapsulating or bonding a drug to its respective carrier, it is also important to consider the ratio of drug:carrier, as this can affect encapsulation/bonding efficiency [71].

In recent years GO has gained a lot of attention for its potential use in biomedical applications, with several publications reporting its benefits as a drug in cancer therapy [72,73,74,75,76,77,78,79,80,81]. For example, Liu et al. [71] investigated a possible nanocarrier for oral drug administration in cancer treatment, developing a thin film with graphene as nanocarrier and pingyangmycin (PYM) as the anti-cancer drug of choice. Orally administered drugs have reduced side effects when compared with IV administration and are more easily accepted by the patients; however, they have some drawbacks, such as being easily degraded by the gastric acid and pepsin present in the stomach, low bioavailability, rapid clearance, and poor tissue distribution. The developed film was composed of poly(acrylic acid)-cysteine (PAA-cys), poly(allylamine hydrochloride) (PAH), and GO as follows: PAA-cys-PAH-GO-PYM. PAA-cys helps the DDS to adhere to the small intestine mucus layer, improving the drug bioavailability, and PAA-cys and PAH polyelectrolytes are cross-linked in the surface of the GO-PYM conjugate and help protect the nanocarrier and drug from gastric acids, allowing the safe passage of the DDS to the intestine, where it can release the drug. Drug release was higher at lower pH, further demonstrating the potential of this DDS in improving targeting to the tumor tissues (acidic environment) and reducing effects in normal tissue.

Another study reports the use of GO NPs as nanocarriers to enhance the drug bioavailability and water solubility. GO is subjected to the “piercing effect” and, therefore, is easily internalized by cells, and its sheet-like structure helps to stabilize the capsule layers, avoiding any drug escape before it reaches its target, making it an ideal choice for this kind of application. Here, they coated GO NPs with a single layer of carboxymethylcellulose (CMC) (to increase drug loading capabilities), with curcumin (Cur), a powerful anti-cancer “drug”, encapsulated into CMC. CMC was then cross-linked with poly N-vinylpyrrolidone (PVP) to produce stimuli-responsive NPs (redox-responsive disulfide linkage). The high glutathione concentrations inside the tumor induce breakage on the bridge between CMC and PVP and, therefore, cause the curcumin to be released. Further NP functionalization was obtained by conjugation of PEGylated monoclonal folic acid antibody (FA) onto the NP surface. This is an important step, as FA binds to folate receptors that are highly expressed in cancer cells, thus helping to direct the NPs to these cells and allowing them to be internalized by phagocytosis. PEGylation helps to reduce protein adsorption onto nanocarriers (preventing “protein corona” and, therefore, renal clearance), prolongs NPs blood circulation, and enhances binding efficiency of FA to the receptors. It also helps to prevent the drug from leaking early and protects it against degradation or enzymatic cleavage. The final composition of the NPs was Cur-FA-CMC/PVP GO NPs. Another important piece of information to retain is that highly charged NPs suffer less aggregation due to repulsive electrostatic forces, allowing for more stable dispersion in physiological conditions [8,61].

Associated with cancer treatment, several stimuli-responsive films which can be used in photothermal therapy are also being studied [61,82,83,84,85]. Photothermal therapy relies on the capacity of NPs to convert NIR (near-infrared radiation) to vibrational energy, generating heat and thus killing cancer cells (where the NPs accumulate). The high efficiency of NIR is mainly because it can reach the NPs without damaging the tissues in between, since biological tissues do not possess chromophores absorbing in the NIR region. GO is a great nanomaterial to use in these kinds of films, as it can generate heat sufficient to kill cancer cells when exposed to NIR, being even more effective than CNT [86]. Therefore, by using GO, it is possible to obtain a NIR-responsive capsule/film with drug loading capabilities and in which GO not only serves as a structural component but also as a NIR responsive material, avoiding the use of gold and silver NPs or addition of NIR-dyes or CNT (which were used until now to fabricate NIR-responsive capsules). This allows for a much simpler and cost-effective process [10].

A study led by Xie et al. [33] successfully developed a magnetic DDS based on GO sheets, with both magnetic and photothermal response. This kind of particle can be used in targeted therapy and has been greatly studied in cancer applications. For this work, Xie et al. [33] developed a graphene oxide-based nanocomposite loaded with doxorubicin (DOX—an antitumor antibiotic). GO has a large DOX loading capacity, making it the ideal nanocarrier. To magnetize the GO sheets, the authors used the thermal reaction method to deposit Fe_3_O_4_ onto the GO sheets. However, mGO (magnetic GO) is even more prone to aggregation than GO, and, therefore, it requires further functionalization. For that purpose, mGO sheets were coated with Chi and SA through the LbL technique. The final composite composition was mGO-Chi/SA-DOX. A ratio of mGO sheets:Chi:SA of 1:4:4 was needed in order to achieve a stable dispersion. DOX was loaded via π-π stacking and electrostatic attraction. They successfully developed a nanocarrier that not only could be directed to the target cells with magnetism, as it had strong photothermal response, causing the cancer cells to die upon NIR irradiation, but had a pH-dependent release of DOX, ideal to use with cancer cells that have a pH lower than normal cells.

Another example is GO-iron oxide (IO) PEGylated nanocomposites (GO-IONP-PEG) that can be used as a drug carrier triggered by a magnetic field, being used in photothermal therapy. GO functionalization with PEG greatly increases its stability in physiological solutions. In these cases, GO-IONP-PEG particles can be loaded, for example, with DOX by π-π stacking and then be used in cancer treatment. However, this composite can also be used as a t2-weighted magnetic resonance contrast in tumors, as demonstrated by Ma et al. [87].

Another potential application for GO-based compounds is as a carrier and/or enhancer for antibacterial or antimicrobial agents [6,76,88,89,90].

As an example, we can use the work carried out by Cao et al. [6], in which they developed a system for sustained release of an antibacterial peptide (G(IIKK)_4_I-NH_2_) using GO as a nanocarrier and assembling it into a thin film that can be used, for example, as coating for surfaces or devices. G(IIKK)_4_I-NH_2_ cannot be efficiently loaded into LbL by itself due to its low charge number, hence the need for a GO. The films were composed of G(IIKK)_4_I-NH_2_ (positively charged), poly(acrylic acid) (PAA, Polyanion), and poly(β-amino ester) (PBAE, polycation), and it was possible to confirm that G(IIKK)_4_I-NH_2_ retained its antibacterial properties even when incorporated in the films. The release speed can be tuned by varying the number of layers of PBAE.

GO’s ability to reach the brain makes it also a good candidate for neurological applications [15,91,92], as reported, for example, by Xiong et al. [91], who developed a DDS to treat Parkinson’s disease (PD) using lactoferrin (Lf)-functionalized GO as a nanocarrier for the natural drug puerarin (Pue), which presents anti-PD properties (Lf-GO-Pue). Lf was used for its ability to bind to the vascular endothelial receptor in the BBB and, therefore, promote the transport of this DDS across this barrier through a receptor-mediated transport. Their work showed promising results, with the in vivo test showing that, in PD-induced mice, there were significantly less neuronal damage and compartmental deficits.

**Figure 3:**
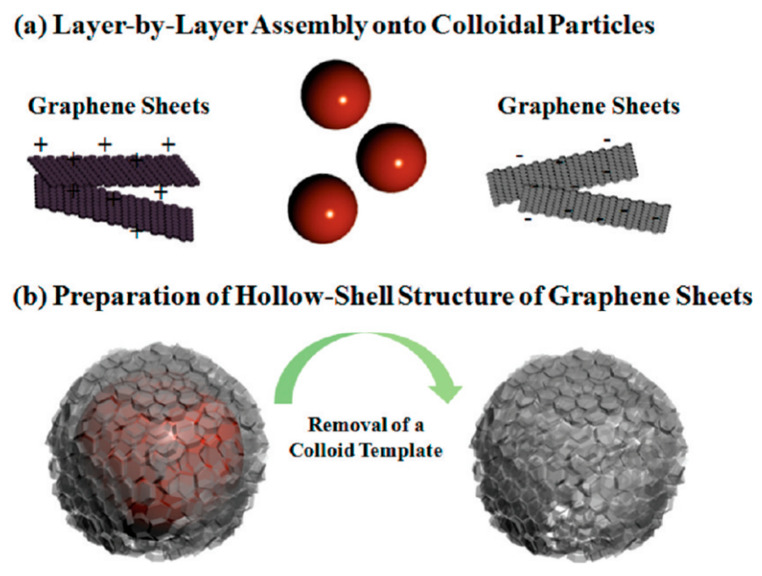
Schematic representation of rGO hollow capsules assembly. Republished with permission from Ref. [3]. Copyright 2022 American Chemical Society.

Furthermore, it is possible to obtain hollow capsules of rGO that can be used to incorporate particles or bioactive molecules. These capsules were obtained by the sequential adsorption (due to electrostatic interactions) of positively charged (rGO-NH_3_^+^) and negatively charged (rGO-COO^−^)) onto a sacrificial PS (polystyrene) substrate, which can then be removed by tetrahydrofuran (THF) solvent exposure (see Figure 3) [3].

Besides the already mentioned examples, there are several others that report using GO as a nanocarrier, for example, for proteins, protecting them against proteolysis and helping retain their activity [93]; as a carrier for pirfenidone (a drug used to treat subarachnoid hemorrhage) [94]; as a carrier for phytomedicines, augmenting their biocompatibility and reducing their toxicity [95]; as a carrier for famotidine (an anti-ulcerous medicine), reducing its side effects through a controlled and sustained release [96]; as a carrier for quercetin (a bioactive flavonoid with powerful antioxidant characteristics), helping circumvent its low bioavailability, extensive first passage metabolism, and instability in aqueous intestinal fluids [97]; as a carrier for transdermal delivery (hydrogel) of ondansetron, a medicine to help control vertigo that has low bioavailability and short half-life [98]; as a carrier for growth factors to enhance cell differentiation [99]; and as a carrier for pain management medicines such as flurbiprofen or buprenorphine, helping reduce its side effects and promoting a sustained release without constant need for dosing [100,101].

## 4. Graphene Oxide for Controlled Drug Release

In a DDS, it is of the utmost importance to control the release of the bioactive compounds. Recently, graphene-based materials, such as graphene oxide, have been gaining a lot of attention for their ability to act as a barrier or capping layer, delaying and controlling the release of biomolecules across time [21,57,102,103,104,105,106].

There are several studies using ovalbumin (OVA—the main protein found in egg white, largely used as a nutrient supplement) as model drug; some authors were able to use GO either as a nanocarrier or a capping layer to build thin films, preventing the early release of the biomacromolecule and obtaining, therefore, a long-term delivery that lasted over 70 days [35]. This mechanism was also applied in films containing OVA as the model protein/drug and PBAE as a hydrolytically degradable polymer. They were able to delay the release of OVA from less than an hour to several weeks by using GO as a capping layer in films with (PBAE/OVA)_20_(GO^+^/GO^−^)_n_, or as a barrier layer in films with (PBAE/OVA-AF555)_20_(GO^+^/GO^−^)_5_(PBAE/OVA-TR)_20_(GO^+^/GO^−^)_2_(PBAE/OVA-FL)_20_ (where AF555—Alexa fluor; FL—fluorescein; and TR—Texas red were used as labels to differentiate between OVAs). When GO was used as a barrier layer, a sequential and controlled release of OVA was possible, with the possibility to control the release gaps by varying the number of GO bilayers. Furthermore, GO’s low permeability helps to reduce interlayer diffusion. This opens up a world of possibilities for DDS with multiple drugs which can be release in a sequential and ordered manner [57].

In an electro-responsive film, GO was also used as a barrier layer. OVA was used as model drug, and rGO was also incorporated in the films as an electroconductive material (because GO has poor conductivity). (PBAE/rGO^−^/GO^+^/OVA/GO^+^/rGO^−^)_n_ (n stands for the number of repeated layers) showed negligible OVA release when there were no stimuli present and 50 times more OVA release upon electric stimulation, further confirming GO barrier capabilities [56].

rGO can also be used as a barrier layer in order to prevent spontaneous release of cDNA, as demonstrated by Jeong et al. [21], who developed a method to activate DNA nanodevices based on electro-responsive films containing rGO with the following composition: (PBAE/cDNA/rGO^+^/cDNA/rGO^+^/PEDOT:PSS)_n_/(rGO^+^/rGO^−^)_n_, where PEDOT:PSS stands for poly(3,4-ethylenedioxythiophene) polystyrene sulfonate.

GO is also able to prevent the release of drugs in multilayered systems. A recent work related with LbL films composed of brimonidine (Brim), an alpha-2 adrenergic agonist used normally to lower intraocular pressure, encapsulated in polycyclodextrins (PolyCD+Brim) intercalated with layers of PBAE and bilayers of charged GO showed that GO can delay the drug release for several days. The study compares films with and without GO (the film (PBAE/PolyCD+Brim)_6_/GO^+^/GO^−^/(PBAE/PolyCD+Brim)_4_GO^+^/GO^−^/(PBAE/PolyCD+Brim)_4_ and the film (PBAE/PolyCD+Brim)_4_), and it was observed that the GO has an important role in the management of drug behavior. The number of GO bilayers is proportional to the delay time, suggesting that this system is a good model to fine-tune a DDS able to deliver a precise drug concentration at a specific period of time [34].

GO can also be used to coat a siRNA-loaded porous silicon (Si) NP in order to delay siRNA release, being able not only to slow down the release time but also the enzymatic degradation of the siRNA and the dissolution of the porous Si NP [107].

## 5. Graphene Oxide: Biocompatible or Cytotoxic?

Graphene biocompatibility is a controversial theme. Whereas some authors report graphene as biocompatible or with no or negligible cytotoxicity, others report it as cytotoxic with the potential to cause damage to living organisms.

Particle size can have an impact on cytotoxicity. Particles with sizes up to 100 nm are able to enter the cell, being able to enter the nucleus below 40 nm. Furthermore, particles below 35 nm are able to cross the BBB. When graphene enters the cell, there is a potential to cause cell damage; this can perhaps be minimized through functionalization [108]. In the majority of cases, coating GO with biocompatible polymers helps improve its biocompatibility and reduce its toxicity [87]. Furthermore, Hu et al. [109] propose that GO cytotoxicity is due to a physical damage to the cell membrane that occurs upon initial contact, and, therefore, it doesn’t depend on time [96,110].

GO functionalization with chitosan allows further biocompatibility, due to chitosan’s antibacterial, antifungal, mucoadhesive, and hemostatic properties and its positive charge, which allows for the functionalized GO to bind with biomolecules with negative charges [7,111].

### 5.1. In Vitro Assays for Graphene Oxide

The first step is to assess GO toxicity, resorting to in vitro testing in various cell lines.

Cytotoxicity is evaluated by incubating films/particles with desired cells in different ratios and then accessing cell viability, for example, through cck-8 assay [61,62], MTT assay [35,70,109], live/dead assay [61], or WST-8 assay [112], among others. Even though several groups report using MTT assay, this is not the ideal test for assessing cytotoxicity of GO, because it reacts with the MTT reagents, directly creating a false-positive cell viability regardless of the cell’s state [112].

Internalization/cellular uptake can be studied by labeling the films or nanocomposites with fluorescein isothiocyanate (FITC) and then staining the nuclei with 4′,6-diamidino-2-phenylindole (DAPI) to study the co-localization using confocal laser scanning microscopy (CLSM) [33,61].

According to Hu et al. [109], protein corona has an impact on GO cytotoxicity. In order to evaluate that, they coated GO with fetal bovine serum (FBS), performed a cell viability assay, and compared the A549 cell’s viability either with GO or FBS-coated GO and concluded that, contrary to GO, which presented a cell viability of around 60%, FBS-coated GO presented a viability of almost 100%, either at 4 or 37 °C.

Several authors demonstrated that GO or rGO-containing films/DDS show no or negligible toxicity to cells, in some cases even at high concentrations [33,34,58,87,91]. Agarwal et al. [70] demonstrated biocompatibility of rGO with rat pheochromocytoma cells (PC12), human oligodendroglia (HOG) cells, and human fetal osteoblast (hFOB) cells. Similarly, Hong et al. [57] detected GO cytotoxicity, but they decided to use one of the most sensitive primary cell types, hematopoietic stem cells (HSCs), with 20% of the cells remaining after 10 days. However, it is important to mention that, besides the high sensitivity of the cells to external perturbations, GO concentrations used were much higher than one would normally expect to use in vivo, and, therefore, this represents a promising outcome.

There are reports that state that GO has a dose-dependent cytotoxicity. Using human dermal fibroblast (HDF) cells, Wang et al. [62], demonstrated that GO in concentrations up to 20 µg/mL shows no toxicity; however, in concentrations of 50 µg/mL, the damage to the cells was obvious, with a growing decrease in cell viability and cell adhesion and with GO being able to enter the cell.

Another study that focused on the dose-dependent cytotoxicity of GO was the one performed by Liao et al. [112]. They were able to prove this dependency; however, even with doses up to 100 µg/mL, the cell viability (human skin fibroblast) remained above 80%. They also accessed GO’s hemolytic activity and verified that it was correlated not only with dosage but also with the particle’s size, being that smaller GO showed increased hemolytic activity. That being said, it is important to mention that GO sheets covered with chitosan sowed no hemolytic activity.

The effect of physical contact of GO with the cell membranes (HDF cells) was also evaluated in the past by placing the Col/GO (collagen) films on the wall of a culture well. They tested two hypotheses, one in which the GO was on the outside, Col/GO, and another one where Col was on the outside, GO/Col, and, therefore, there was no direct contact of GO with cell membrane. Their results show a decrease in cell viability with the films with GO on the outside, but it remained above 80% [35].

Taking into consideration the reports presented here, it is clear that further research regarding GO toxicity is still required, but the majority of the studies seem to suggest GO has a negligible toxicity, demonstrating that it is safe to use in biomedical applications [113].

### 5.2. In Vivo Assays for Graphene Oxide

GO in vivo toxicity is not well described yet, but we can nonetheless find some reports on this subject. In vivo assays (mice) of Lf-GO-Pue DDS show that after 8 days of treatment there are no differences either in the blood panel or in histological analysis of major organs between controls and treated mice [91].

Wang et al. [62] obtained results in mice somewhat similar to what they had already observed in vitro, i.e., that in concentrations up to 0.25 mg (administered by tail injection) there was no cytotoxicity, but in doses of 0.4 mg the toxic effects of GO are apparent, causing chronic toxicity, lung granuloma, and even death of some animals caused by GO agglomeration in the mouse airways leading to suffocation. The kidney’s inability to clear GO from the system, perhaps due to its sheet-like structure, may hamper its biomedical applications.

In another study, mice with 5 consecutive days of high rGO doses administered orally (300 mg/kg for small nanosheets and 60 mg/kg for large nanosheets) showed no lasting alterations in locomotory activity, neuromuscular coordination, behavior, anxiety, and memory or at a physiological level. The mice did show reduced energy and locomotor activity in the first days of treatment; these effects disappeared after 15 or 60 days. This can be due to the abnormal accumulation of rGO particles in the organs during the first days that tend to disappear with time [114].

That being said, there are several authors who report the harmful effects that GO can have in several models; namely, Fu et al. [115] reports the damaging effects to mice offspring in the lactation period; Chen et al. [116] and Dasmahapatra et al. [117] reported the toxicity GO poses to fish, especially when it is “released” into the environment, either during embryogenesis or adulthood; and Guo et al. [118] reported these effects in W1118 flies.

There are still few studies regarding GO toxicity in vivo, and further studies are required. It would be especially interesting to have reports about its toxicity in non-human primates before we can definitely conclude that it is safe. However, the reports existing so far look promising [119,120].

## 6. Graphene Oxide in Biomedical Applications

GO has many potential applications in the biomedical fields as, for example, part of wound-healing membranes or dressing patches [121,122,123]; as non-viral gene transfer vectors [124,125]; as part of oxygen delivery systems [126]; as part of mosquito-bite protective fabrics [127]; as a substrate to graft polymer brushes in order to obtain scaffolds that can be used in cell proliferation and tissue engineering [128]; as coating for biomedical implants [129,130,131]; as bioactive components or scaffolds in bone tissue engineering/regeneration [132,133,134]; as scaffolds to drive neuronal growth and regeneration [92]; as parts of biomimetic interfaces for monitoring cell behaviors [135]; as a therapeutic anti-angiogenic agent [136]; as a component in contact lenses to help corneal epithelial healing [137]; as part of a self-healing hydrogel capable of slow release of lubrification for artificial joints [138]; as part of free-standing films with applications in tissue engineering and wound healing [111]; or even as part of biosensors to track non-communicable diseases [139].

For example, it is possible to functionalize GO using synthetic peptides (Pep), with osteogenic or neurogenic capabilities, forming a Pep-GO conjugate which is biocompatible and electro-conductive, and which can be used either as scaffolds or coating for biomedical devices. These conjugates have the ability to promote, for example, cell adhesion and proliferation, neurogenesis, or osteogenesis. This type of functionalization (contrary, for example, to PEG, dextran, or poly(acrylic) acid functionalizations, which are inert and do not promote healing) can instruct the cells towards healing using biochemical cues. These functional graphenic materials (FGMs) are obtained by covalently functionalizing GO with bioactive moieties. For this work, Claisen graphene (CG) was used for its enhanced conductivity and biocompatibility when compared with normal GO. GC is a reduced GO with several carboxylic acids in the surface and edges of the sheets. As for peptides, polyglutamate (p(glu)) and polylysine (p(lys)) were used for their osteogenic and cellular adhesion enhancement properties, respectively [140].

Another example of a coating for biomedical devices is the work reported by Gao et al. [7] which reported a chitosan-functionalized GO (GOChi)/Heparin (Hep) coating for a biodegradable magnesium alloy stent to improve its corrosion resistance and biocompatibility. These stents can be used as interventional therapy to treat cardiovascular disease and, therefore, minimize inflammatory reactions and in-stent restenosis, which occurs with metal stents.

Microfluidic immunoassay devices are another possibility, as reported by Miyazaki et al. [141], who developed a device based on poly(methylmethacrylate) (PMMA) coated/functionalized with a thin film of poly(ethylene imine) (PEI) and GO ((PEI/GO)_5_). The antibody of choice was anti-rat-IgG. This coating enhanced the PMMA’s wettability (which can influence protein adsorption, blood coagulation, and platelet, cell, and bacterial adhesion), hydrophilicity, and roughness, which helps the adsorption of the antibodies. Therefore, they were able to improve the antibody binding efficiency, reducing the time of incubation from 2 h to 15 min, when compared to uncoated PMMA, to obtain a good surface coverage.

Some examples of biosensors are, for example, the work done by Kumarasamy et al. [69] and Mascagni et al. [65]. The first one developed an ultrasensitive DNA hybridization sensor using a one-step (simultaneous electro-reduction of both graphene oxide and gold chloride and co-deposition techniques) electrodeposition-assisted LbL technique to obtain it. A glassy carbon electrode (GCE) was coated with gold NPs (AuNPs) and rGO. The AuNP/rGO/AuNP/GCE composite with 3D nanoarchitecture had self-healing properties and was used to immobilize DNA onto its surface. This can be useful, for example, for cancer diagnosis. In its turn, Mascagni et al. [65] demonstrated the GO potential to be used in biosensors, as they successfully developed a film capable of sensing glucose. This potential is mainly due to the high electrical conductivity of graphene oxide and its ability to maintain the biocatalytic activity of the enzymes used to sense glucose.

Tissue engineering is also an emerging field in which GO has been widely used. Shin et al. [11], for example, presented a method based on GO-PLL particles and the LbL technique particles to construct 3D tissue structures with cardiac cells that presented spontaneous beating behavior and pumping capabilities mimicking the native heart tissue. GO showed to improved cell adhesiveness, facilitating cell separation and stacking while allowing the passage of oxygen and nutrients.

As mentioned before, stimuli-responsive devices have a huge part to play in future biomedical applications. Deng et al. [61] developed a hybrid microcapsule (h-MC) which was capable of responding to two external stimuli (magnetic field and NIR laser) when they were simultaneously applied, augmenting, therefore, the ability to control drug release (DOX, in this case). The microcapsules are formed by layer-by-layer using iron oxide-decorated graphene oxide (Fe_3_O_4_@GO) and SA, Chi, and hyaluronic acid (HA), which are deposited onto a sacrificial template (monodisperse spherical HCL-soluble melamine formaldehyde resin particles). The graphene capsules can then be loaded with DOX through pH control. Chi and Alg are biocompatible and respond to both pH and temperature stimuli. HA has a great biodistribution in cancer cells. Polysaccharides in general are able to interact with cell membranes, promoting their internalization. The authors have successfully developed a stimuli-responsive h-MC (SA/Chi/Fe_3_O_4_@GO/Chi/HA) with negligible cytotoxicity that can be used as hyperthermia therapy.

**Figure 4:**
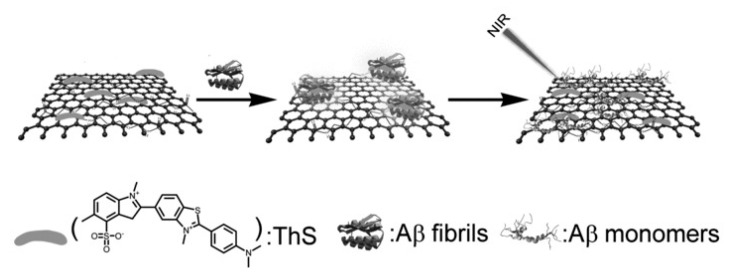
Schematic representation of the GO-ThS system used for AD treatment. Upon NIR irradiation, the GO-ThS system can dissolve the Aβ amyloid deposits. Republished with permission from Ref. [15]. Copyright 2022 John Wiley and Sons.

**Figure 5:**
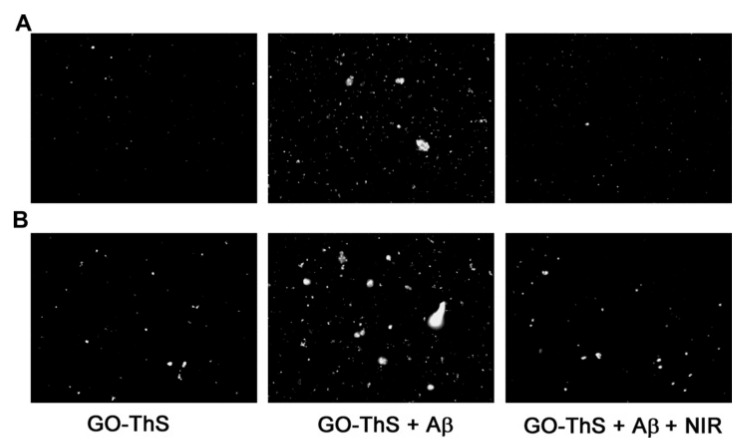
Fluorescence images of Aβ incubated with GO-ThS in Tris buffer (**A**) and mice CSF (**B**) (200x magnification) demonstrate that upon NIR irradiation GO-ThS can effectively dissociate Aβ amyloid deposits. Republished with permission from Ref. [15]. Copyright 2022 John Wiley and Sons.

Even though the vast majority of reports related to stimuli-responsive devices are in the oncology area, there are some reports in other fields of medicine, such as Li et al. [15], which harnessed GO’s strong NIR absorption and hyperthermic effects in order to develop a conjugate which can be used to treat Alzheimer’s disease (AD). They used carboxyl-modified GO (GO-COOH) and functionalized it by covalent interactions with diaminotriethylene glycol to obtain amino-functionalized GO (AGO). After that, the AGO conjugated with activated ThS (thioflavin-S) to obtain GO-ThS. ThS can selectively bind to Aβ fibrils, and GO has the ability to pass the BBB and can effectively generate heat upon NIR irradiation and, therefore, promote the dissociation of Aβ fibrils (Figure 4). NIR spatial precision and capacity to penetrate tissues makes it a great asset in targeted therapy, because it avoids damage to surrounding tissues. The author proved that both in PC12 cells and in cerebrospinal fluid (CSF) of mice that GO-Ths, upon NIR irradiation, can effectively dissociate Aβ plaques/fibrils (which are the pathological hallmarks of AD), making it a great candidate for AD photothermal treatment. Moreover, Aβ dissociation can be monitored through changes in ThS fluorescence upon binding with these fibrils (Figure 5). Silva et al.’s [142] work is another example. They showed that nanographene oxide (GOn—average sizes of 197.6 ± 11.8 nm) can permeate the skin, with no cytotoxic effects. GOn dispersions are able to absorb NIR radiation and can, therefore, be used to apply local photothermal therapy or as DDS to treat skin conditions.

With the increasing rise in antimicrobial resistance across the globe, there is an urgency in find alternatives to the use of antibiotics, and, therefore, GO has also been studied in this context. One example is the work developed by Wu et al. [143], which demonstrated that GO alone does not show antimicrobial activity and can in fact promote bacterial growth and attachment; however, GO-based materials such as GO-polyoxyalkyleneamine (POAA) (in concentrations over 50 µg/mL) and GO-Chi can be used with success as microbial agents, as they reduced bacterial viability when added to the bacterial colonies. Another example is Katuwavila et al.’s [144] work that showed that cephalexin (CEF), a broad-spectrum antibiotic, antibacterial activity could be enhanced and more sustained when CEF was loaded into PEGylated GO (GO-PEG-CEF).

As stated throughout this chapter, there have been several studies regarding GO applications in biomedical devices [145] in the last few years, proving its great potential in this field. Its physical and chemical properties allied with the versatility of the layer-by-layer technique allow for an easy and cost-effective way to develop DDS applied to the most varied biomedical devices.

## 7. Conclusions

Graphene oxide is a versatile material that has been emerging in the biomedical field in the last few years, mainly due to its physical and chemical properties. Furthermore, the simple self-assembly method of LbL allows for an easy, quick, and cost-effective process that allows large-scale production. As stated throughout this review, there are numerous applications which can benefit from GO’s incorporation either as a carrier, due to the large amounts of drug it can encapsulate and transport (even across the BBB), or as a structural component maintaining the integrity of the DDS until they reach their target, preventing leakage of bioactive compounds, or as a barrier or capping layer allowing for the fine-tuning of this DDS and promoting a sequential and time-controlled release of biomolecules. However, there are several challenges ahead, and further research in this field is still necessary. Interdiffusion between layers, even when GO is used, remains a problem, and further studies are needed in order to really understand how to fine-tune the sequential release of drugs over long periods of time. The controversial results obtained in the cytotoxic studies also need further enlightenment, as there are few studies clearly stating GO toxicity in vivo, especially long-term, and this needs to be addressed for the proposed applications to be used as day-to-day tools in patient care. Furthermore, even though GO’s potential for biomedical applications still requires further evaluation, new and promising applications are being studied every day.

## Figures and Tables

**Table 1:** Compilation of different techniques used to characterize the assembly of LbL films which contain GO.

Technique	Purpose
UV-Visible/Fluorescence Spectroscopy	GO structure [58]
Atomic Force Microscopy (AFM)	Surface morphology and roughness [21,32,34,35,57,58,59,60]
Scanning Electron Microscopy (SEM)	Surface morphology [21,35,36,54,55,56,57,58]
Transmission Electron Microscopy (TEM)	Surface morphology [36,37,61] GO structure [62]
Dynamic Light Scattering (DLS) method	Zeta potential and particle size and distribution [36,37,55,61]
Quartz Crystal Microbalance (QCM)	Layer adsorption [21,34,35,54,63]
Profilometry	Layer thickness [56,64]
Raman Spectroscopy (RS)	Layer deposition [61] GO structure [58]
SQUID—Field-dependent magnetization measurement	Magnetism measurement [61]
Fourier Transform Infrared Spectroscopy (FTIR)	GO structure [58]
Surface Plasmon Resonance (SPR)	Film growth [65]
Scanning Tunneling Microscopy (STM)	Characterization at molecular scale [66,67]
X-ray Diffraction	GO structure [33,58,68,69] Interlayer space [65]
X-ray Photoelectron Spectroscopy (XPS)	Film chemical characteristics [69,70]

## Data Availability

Not applicable.

## Abbreviations

AD	Alzheimer’s Disease
AF555	Alexa Fluor
AFM	Atomic Force Microscopy
AGO	Amino-functionalized GO
APTES	((3-aminopropyl) triethoxysilane)
AuNPs	Gold Nanoparticles
BBB	Blood Brain Barrier
Brim	Brimonidine
CD	Circular Dichroism
CEF	Cephalexin
CG	Claisen Graphene
Chi	Chitosan
CLSM	Confocal Laser Scanning Microscopy
CMC	Carboxymethylcellulose
CNT	Carbon Nanotubes
CSF	Cerebrospinal Fluid
Cur	Curcumin
DAPI	4′,6-diamidino-2-phenylindole
DDS	Drug Delivery System
DI	Deionized
DLS	Dynamic Light Scattering
DOX	Doxorubicin
FA	Folic Acid Antibody
FBS	Fetal Bovine Serum
Fe3O4@GO	Iron Oxide decorated Graphene Oxide
FGMs	Functional Graphenic Materials
FITIC	Fluorescein Isothiocyanate
FL	Fluorescein
FTIR	Fourier-Transform Infrared Spectroscopy
G(IIKK)4I-NH2	Antibacterial peptide
GCE	Glassy Carbon Electrode
GO	Graphene Oxide
GOChi	Chitosan functionalized Graphene Oxide
GO-IONP	Graphene Oxide—Iron Oxide nanocomposites
GOn	Nano Graphene Oxide
HA	Hyaluronic Acid
HDF	Human Dermal Fibroblast
Hep	Heparin
hFOB	Human Fetal Osteoblast
h-MC	Hybrid Microcapsule
HOG	Human Oligodendroglia
HSCs	Hematopoietic Stem Cells
IO	Iron Oxide
LbL	Layer-by-layer
Lf	Lactoferrin
mGO	Magnetic Graphene Oxide
NIR	Near Infrared Radiation
NP’s	Nanoparticles
OVA	Ovalbumin
P(glu)	Poly glutamate
P(lys)	Poly lysine
PAA	Poly (acrylic acid)
PAA-Cys	Poly(acrylic acid)-cysteine
PAH	Poly(allylamine hydrochloride)
PB	1-Pyrenebutyrate
PBAE	Poly(β-amino ester)
PBS	Phosphate Buffered Saline
PC12	Pheochromocytoma Cells
PD	Parkinson’s Disease
PEDOT:PSS	Poly(3,4-ethylenedioxythiophene) polystyrene sulfonate
PEG	Polyethylene glycol
PEI	Poly(ethylene imine)
PEMf	Polyelectrolyte Multilayer films
Pep	Synthetic peptides
PLL	Poly L-Lysine
PMMA	Poly(methylmethacrylate)
POAA	Polyoxyalkyleneamine
PolyCD	Polycyclodextrins
PS	Polystyrene
Pue	Puerarin
PVP	Poly N-vinylpyrrolidone
PYM	Pingyangmycin
QCM	Quartz Crystal Microbalance
rGO	Reduced Graphene Oxide
RS	Raman Spectroscopy
SA	Sodium Alginate
SEM	Scanning Electron Microscopy
Si	Silicon
SPR	Surface Plasmon Resonance
STM	Scanning Tunneling Microscopy
TEM	Transmission Electron Microscopy
THF	Tetrahydrofuran
ThS	Thioflavin S
TR	Texas Red
XPS	X-ray Photoelectron Spectroscopy
